# Asymmetry in Muscle Activation and Co-Contraction Between Lower Limb During Zap-3 Flamenco Footwork

**DOI:** 10.3390/s25154829

**Published:** 2025-08-06

**Authors:** Ningyi Zhang, Sebastián Gómez-Lozano, Ross Armstrong, Hui Liu, Ce Guo, Alfonso Vargas-Macías

**Affiliations:** 1 Performing Arts Research Group, Faculty of Sport, San Antonio Catholic University, 30107 Murcia, Spain; nzhang@alu.ucam.edu (N.Z.); sglozano@ucam.edu (S.G.-L.); 2 Telethusa Centre for Flamenco Research, 11004 Cádiz, Spain; vargas@flamencoinvestigacion.es; 3 Rehabilitation and Healthy Lives Research Group, Institute of Health, University of Cumbria, Carlisle CA1 2HH, UK; ross.armstrong@cumbria.ac.uk; 4 Biomechanics Laboratory, Beijing Sport University, Beijing 100084, China; liuhui@bsu.edu.cn; 5 School of Athletic Performance, Shanghai University of Sport, Shanghai 200438, China

**Keywords:** surface EMG, musculoskeletal, dance, injury, asymmetry

## Abstract

This study aims to investigate asymmetries in muscle activation and co-contraction of main lower limb muscles during flamenco Zap-3 footwork with consideration of the footwork speed and dancer proficiency. Twelve flamenco dancers participated, including six professionals and six amateurs. Each participant performed the Zap-3 sequence under three speed conditions: 160 beats per minute (bpm), 180 bpm and the fastest speed level (F). The normalized surface electromyography was recorded in the gastrocnemius medialis (GM), biceps femoris (BF), tibialis anterior (TA) and rectus femoris (RF) in the dominant (DL) and non-dominant leg (NDL). The co-contraction index was also calculated for selected muscle pairs. The results showed that significant asymmetries occurred only in professional dancers and exclusively at the F speed level. Specifically, the value of the GM in the NDL was higher than that of the DL (*p* < 0.05, d = 1.97); the value of the BF in the DL was higher than that of the NDL (*p* < 0.05, d = 1.86) and the co-contraction index of BF/RF in the DL was higher than that of the NDL (*p* < 0.05, d = 1.87). Understanding these asymmetries may help to inform individualized training strategies aimed at optimizing performance and reducing potential risks.

## 1. Introduction

Flamenco footwork techniques place significant physical demands on dancers [1] who use various parts of the feet, including the heel and toe tips, to strike the floor and create rhythmic, loud sounds that produce strong vibrations [2]. These vibrations travel from the joints of the lower limbs to the spine, potentially causing pain and overuse injuries that can significantly impact the development of a dancer’s career, potentially leading to their replacement by healthier individuals [3,4,5,6]. Additionally, such injuries can affect their daily life and psychological well-being [7].

Previous research has demonstrated that flamenco dancers experience a high incidence of injuries, with one of the most frequently affected areas being the lower limbs [4,8]. Factors contributing to these injuries, in addition to demographic characteristics, also include the dancer’s level of proficiency [9]. Studies have indicated that professional dancers and athletes face a higher frequency of injuries compared to students and amateurs, and injury risk factors such as balance performance and ankle active range of motion affect amateur and professional flamenco dancers differently [10]. Therefore, when studying flamenco dancers, it is essential to examine these levels of dancers, namely, professional and amateur, separately.

One of the most studied footwork technique sequences, Zap-3, consists of six steps involving one foot, with rapid alternation between heel and toe strikes with the other foot, striking the floor with ball of the foot and with the heel. After completing one sequence, the dancer repeats the next sequence with the opposite foot, maintaining stability in the upper body and trunk throughout the movement. These movements involve not only a sequence of muscle activities necessary for performance but also the regulation of force and speed [11]. Proper execution of these movements depends on meeting specific biomechanical requirements, with electrical signals transmitted through nerves to activate the appropriate muscle groups [12]. In the execution of flamenco footwork, muscular organization plays a critical role in stabilizing the body and mitigating bending stresses on the skeletal system. It also serves to attenuate peak dynamic loads and vibration, thereby minimizing the risk of damage to musculoskeletal tissues [13].

Electromyography (EMG) is a method dedicated to the development, recording and analysis of myoelectric signals [14,15]. Surface EMG (sEMG) has been extensively employed in different dance genres such as ballet [16] to investigate movement characteristics, enhance understanding of the coordinative structure underlying complex dance movements and analyze the neuromuscular independence of the abdominal muscles in trained dancers [17], and the technique has also been employed to study belly dance [18], Argentine tango [19], contemporary dance [17] and sport dance [20]. Muscle co-contraction is recognized as an effective strategy to safeguard joints from potentially hazardous loads [21]. A previous study indicated that a higher CCI in the trailing leg has been observed in injured dancers, suggesting that greater muscular effort is required to ensure ankle stability and maintain postural control [22]. Although sEMG is widely used in research related to dance and other physical activities, it still has certain limitations, such as its inability to directly quantify muscle force or joint loading. However, this study offers preliminary insights into lower limb muscle activation during Zap-3 footwork and contributes to the improvement of techniques and injury prevention to some extent.

To the best of our knowledge, only one study has recorded the activity of the lower limbs during flamenco footwork or in percussive dances using EMG, and this was for an individual professional dancer [11]. The study identified patterns of changes in amplitude and coordination of muscle activity, indicating higher activity in the proximal muscles and somewhat reciprocal activation of antagonists and agonists for maintaining ankle and knee positions [11]. Therefore, this study further explored the asymmetry in muscle activation and co-contraction between lower limb, which is important for investigating the risks of injuries or pain [23,24]. An incorrect adaptation of technique to compensate for anatomical variations can indirectly contribute to dancer injuries. Muscle imbalances or asymmetry may lead to significant injuries due to deficiencies in technique and in both strength and flexibility [24]. However, muscle imbalance or asymmetry remains unexplored in flamenco dancing research.

It is essential to provide medical practitioners, coaches and dancers with a theoretical basis for the effective management of training programs for flamenco dancing to reduce injury risk in accordance with the proficiency levels of dancers. To better inform practical applications and training recommendations, this study aims to investigate asymmetries in muscle activation and co-contraction of major lower limb muscle groups during flamenco Zap-3 footwork. The specific objectives were as follows:

(1) To explore differences in normalized sEMG values between the dominant and non-dominant legs for the gastrocnemius medialis (GM), biceps femoris (BF), tibialis anterior (TA) and rectus femoris (RF) muscle groups.
(2) To evaluate asymmetries in the co-contraction index between the dominant and non-dominant legs for selected muscle pairs (GM/TA and BF/RF).
(3) To perform separate analyses for each speed condition: 160 beat per minute (bpm), 180 bpm and the fastest speed level (F) and dancer group: professional (PRO) and amateur (AMT) dancers.

## 2. Materials and Methods

### 2.1. Participants

The recruitment of participants for this study took place from 30 March to 30 June, 2022. The experimental testing was conducted between 6 July and 20 July, 2022. Twelve female flamenco dancers volunteered in this study, namely, 6 PRO and 6 AMT dancers. A sample size estimation was conducted using G Power 3.1 [25]. Given the study design (paired *t*-tests, α = 0.05, power = 0.90) and the available sample size (n = 6), the minimum effect size was d = 1.67. Descriptive characteristics of the participants are presented (Table 1). The dancers provided informed consent in writing before the commencement of the study and after the experimental procedures, risks and benefits of participation had been explained. Ethical approval was granted by the Sports Science Experiment Ethics Committee of Beijing Sport University (2022037H), and the study was completed in accordance with the Declaration of Helsinki. The inclusion criteria for the PRO group were that participants were professional flamenco dancers who received paid work for teaching, rehearsing or performing in the flamenco dance field and who primarily considered themselves to be professional flamenco dancers with a minimum of 3 years’ experience. For the AMT group, participants were amateur flamenco dancers who engaged in dance for recreational purposes only and attended flamenco dance training for at least 3 h per week and had at least 1 year of flamenco dance experience. Before the study, participants completed a self-reported questionnaire. Exclusion criteria included individuals under 18 years of age, those with less than 1 year of flamenco dance experience and participants who reported heart disease, pregnancy or musculoskeletal injuries within the 6 months prior to the study. None of the participants reported a diagnosis of Ehlers–Danlos syndrome, Marfan syndrome or osteogenesis imperfecta.

### 2.2. Procedures

This research comprised a cross-sectional study. Before testing, all participants were informed regarding the experimental methods and procedures, which were demonstrated by one of the researchers before testing. Muscle activation was measured while participants were performing Zap-3 flamenco footwork. Zap-3 is a tapping footwork sequence, and different types of flooring may potentially influence muscle activation measurements; therefore, each participant was required to stand on the same dance board (92 × 100 cm, wooden floor, specifically designed for flamenco dancing) and perform in a dance studio at 160 bpm, 180 bpm or the fastest (as fast as they can) speed level in sequence (Table 2). Each level was performed 3 times for a duration of 15 s. At 160 bpm and 180 bpm, participants were required to dance with earphones, through which a metronome was played. At the fastest level, they performed as quickly as possible and with a melodic, rhythmic sound. Participants could practice before data recording to allow adaptation to the next speed level (For the F speed level, participants were encouraged to perform the footwork at increasingly faster speeds as long as the movements remained technically correct, until an error occurred). If any participant experienced physical discomfort or symptoms of distress (such as excessive fatigue or muscle discomfort) during the testing, the experiment was immediately stopped to ensure the safety and well-being of the participant. All participants were instructed to wear flamenco footwear similar to that worn during training/performance. Participants were allowed to rest during tests for 3 min between each Zap-3 test. Muscle activation during the footwork was evaluated in the GM, the BF, the TA and the RF muscle groups in the dominant and non-dominant legs.

**Figure 1:**
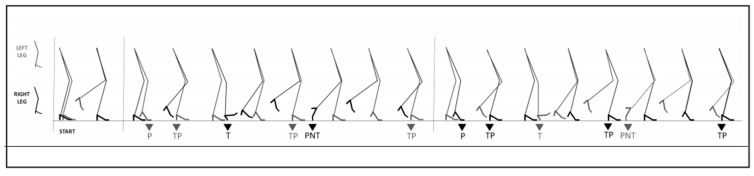
Elaboration of the graphic sequence of the Zap-3 test. zapateado de planta (P), zapateado de tacón–planta (TP), zapateado de tacón (T), zapateado de tacón–planta (TP), zapateado de punta (PNT), zapateado de tacón–planta (TP).

Participants had to perform Zap-3, a sequence of 6 footwork steps with the right and the left foot. When one sequence was completed, they repeated the next sequence with the other foot and repeated alternately with each foot [26,27]. An elaboration of the graphic sequence of the Zap-3 Test is shown in Figure 1, adopted from the Alfonso Vargas-Macías research group [2]. During the entire footwork movement, participants were required to maintain an akimbo posture with their hands to stabilize the upper limbs and trunk and to perform smooth and coherent movements. Two professional flamenco instructors (each with over five years of teaching and performance experience) supervised each trial. If incorrect striking rhythm or sound, foot placement errors or visible instability in the upper body occurred, the trial was deemed invalid and repeated after sufficient rest.

### 2.3. Data Processing

The activation of the muscles was recorded using the Delsys EMG system (Trigno Wireless EMG System, Delsys, Natick, MA, USA), which is compatible with the EMGworks Acquisition 4.7.9 software. Before sensor attachment, the skin was shaved and cleaned with isopropyl alcohol. Then the sensors were attached directly to the skin using medical tape and secured using elastic bandage by a laboratory technician with five years of experience and training in the use of the Delsys EMG system, housed at the GM (placed on the most prominent bulge of the muscle, 1cm distal to the mid-belly of the GM), BF (placed at 50% on the line between the ischial tuberosity and the lateral epicondyle of the tibia), TA (placed at 1/3 on the line between the tip of the fibula and the tip of the medial malleolus) and RF (placed at 50% on the line from the anterior spina iliaca superior to the superior part of the patella) in the dominant and non-dominant leg. The dominant leg was determined as the leg most often used by the participant to kick a ball [28].

The EMG signal was sampled at a frequency of 1000 Hz and then filtered (bandwidth: 15–500 Hz). EMGworks Analysis software was used to process the EMG signal. Related artefacts and noise were inspected visually. The EMG signal was full-wave-rectified and smoothed using the root mean square algorithm with a 50-milisecond window. We normalized the data using mean and peak values of EMG muscle amplitudes according to the following formula: EMG normalized = (EMG mean/EMG peak values) × 100%. Co-contraction index (CCI) = antagonist/agonist, GM/TA; BF/RF were calculated for both the dominant and non-dominant leg [29].

### 2.4. Statistical Analysis

All data were analyzed using a statistical software package (SPSS IBM Statistics V21.0, IBM, Armonk, New York, USA) with descriptive statistics presented as mean ± standard deviation. The normality of the distribution of variables and homogeneity of variance were checked using the Kolmogorov–Smirnov test and Levene’s statistic. The unpaired sample *t*-test and Mann-Whitney U test were used to analyze differences between groups in participants’ age (years), height (meters), mass (kilogram), BMI (kilogram/meter^2^) and flamenco dance experience (years). To explore the bilateral asymmetry (dominant leg vs. non-dominant leg), a paired sample *t*-test was performed, and 95% Confidence Intervals (CIs) are presented for significant findings. The minimum effect size was d = 1.67. This value was used as the threshold for identifying lower limb asymmetries. Differences were deemed statistically significant at the *p* < 0.05 level.

## 3. Results

### 3.1. Normalized Surface Electromyography

The muscle activation of both the dominant and non-dominant leg during the Zap-3 flamenco footwork in PRO and AMT dancers in the GM, BF, TA and RF is presented in Table 3, with the EMG signal normalized as a percentage of the mean/peak EMG values. The results demonstrated there were no significant bilateral asymmetries in the AMT group, but a significant difference was observed in the PRO group. The value of the GM in the non-dominant leg was higher than that of the dominant leg at the F speed level (non-dominant CI: 5.23–8.67; dominant CI: 2.55–5.99, *p* = 0.030 < 0.05; d = 1.97 > 1.67), and the value of BF in the dominant leg (CI: 6.03–9.46) was higher than non-dominant leg (CI: 2.76–6.20, *p* = 0.025 < 0.05; d = 1.86 > 1.67) at the F speed level (Table 3).

### 3.2. The Co-Contraction Index

The muscle activation of both the dominant and non-dominant leg in PRO and AMT dancers in the GM/TA and BF/RF is presented in Table 4. For the CCI, the results demonstrate for the PRO group that the CCI of BF/RF in the dominant leg (1.14 ± 0.33, CI: 0.88–1.40) was higher than that of the non-dominant leg (0.62 ± 0.15, CI: 0.36–0.87, *p* = 0.023 < 0.05; d = 1.87 > 1.67) at the F speed level.

## 4. Discussion

Flamenco dancers experience a high injury risk, with such injuries most frequently occurring in the lower limbs, lumbar and cervical vertebra [4,8]. In the execution of flamenco footwork, muscular organization plays a critical role in stabilizing the body and mitigating bending stresses on the skeletal system. It also serves to attenuate peak dynamic loads and vibration, thereby minimizing the risk of damage to musculoskeletal tissues [13]. Existing research has indicated higher sEMG in the proximal muscles and somewhat reciprocal activation of antagonists and agonists for maintaining ankle and knee positions [11]. Therefore, further research exploring muscle activation, co-contraction and asymmetry is important for optimizing performance and reducing potential risks.

### 4.1. Roles of Lower Limb Muscles During Zap-3 Footwork Execution

During the execution of the Zap-3 movement, which requires rapid alternation of foot strikes with both legs, the rectus femoris (RF) plays a central role in supporting knee joint stability and coordinating transitions between the upper and lower body segments [11,30]. Given that the knee remains in a moderately flexed position throughout the sequence, maintaining controlled muscle activity around this joint is important to minimizing variations in shear forces and reduce the risk of mechanical overload [31,32]. The RF may also contribute to stabilizing the hip joint, which is essential for maintaining balance during dynamic footwork as coordinated control of the hip is necessary for effective transmission of force between the trunk and the lower extremities. Inadequate or poorly modulated RF activity during rapid sequences could compromise this coordination and place additional stress on the knee joint [29]. The tibialis anterior (TA), as the primary dorsiflexor, contributes to ankle joint stabilization and the controlled lifting of the forefoot during each strike. This function is particularly relevant in fast transitions, where rapid dorsiflexion enhances the precision of foot contact with the floor, helping the dancer maintain rhythm and balance. The gastrocnemius medialis (GM), responsible for plantarflexion and contributing to knee flexion, may have a less prominent role during certain steps of Zap-3, where rapid forward foot motion and stable support take precedence over vertical heel elevation. This reflects a shift in muscular demand toward joints and muscles that facilitate speed and precise control, such as the RF and TA. However, adequate GM activation remains important for ankle stability; insufficient control around the ankle joint could increase the risk of imbalance or injury during high-speed execution.

### 4.2. Co-Contraction Index

Muscle co-contraction refers to the concurrent activation of an agonist muscle with its antagonist or synergistic muscles within a specific time frame. This co-contraction reflects the coordination between antagonistic and agonistic muscles and is a key factor influencing muscle contraction strength [33] and may enhance joint stiffness as well [34].

Although there has been no previous research on the synergistic function of lower limb muscles in flamenco dance, we can draw parallels from studies conducted in ballet. One study examined the neuromuscular and biomechanical characteristics of injured and uninjured dancers during the landing phase of the Sissonne Fermée task, revealing that injured dancers exhibited a greater co-contraction index in the non-dominant ankle and a lower loading rate [22]. Furthermore, another study assessed a group of dancers before and after completing a dance-specific fatigue protocol (high-intensity dance performance test) and measured the activation levels of the quadriceps and hamstrings during landings [35]. This study found a significant increase in the co-contraction rates of both muscle groups before and after fatigue. These findings suggest that increased co-contraction indices may reflect a compensatory neuromuscular strategy to enhance joint stability, particularly under conditions of fatigue or instability.

### 4.3. Asymmetry Between Lower Limb

Our study indicated that the sEMG of the GM and BF and the CCI of BF/RF were all different between the dominant and non-dominant leg for the PRO group. The activation of muscles in the dominant leg and their co-contraction with proximal muscles play a crucial role in controlling knee flexion and extension and maintaining lower limb stability. In the Zap-3 movement, the dominant leg must bear a greater load and generate higher force output [6]. This likely reflects a greater reliance on proximal muscle coordination in the dominant leg to support its primary role in force generation and postural support. Conversely, the non-dominant leg may rely more on distal muscle coordination to compensate for rapid directional changes and maintain postural equilibrium during transitions.

In the present study, all significant asymmetries were only observed at the F speed level. This may be attributed to the increased external loads that occur during high-speed execution and the greater influence of lower limb balance ability on load distribution in professional dancers under such conditions. Previous studies have reported a significant main effect of speed on external load during the Zap-3 footwork, indicating that higher speed was associated with elevated mechanical demands [6]. Moreover, another study demonstrated that the relationship between lower limb balance ability and external load was evident only at the fastest execution speed of Zap-3, particularly in professional dancers [10]. These findings suggest that neuromuscular asymmetries may emerge as functional adaptations to the increased biomechanical and postural demands encountered at higher speeds.

Additionally, the effect of lower limb balance asymmetry on external load during flamenco Zap-3 was different between PRO and AMT dancers. Previous research indicated that reducing the difference between bilateral lower limbs on balance ability could be favorable for AMT. However, for PRO dancers the findings were conflicting, and bilateral asymmetry could reduce the external load at the L5 position [10]. This may be because, for PRO dancers, less asymmetry or more stability may cause them to strike the floor with greater force and make a louder sound, thus producing more vibration. This could account for the observed asymmetry in muscle activation and co-contraction between the lower limbs to some extent. In this study, PRO and AMT groups were analyzed separately. While there were no statistical comparisons between groups, it is recognized that professional dancers may demonstrate distinct coordination patterns under faster performance conditions since the asymmetry only appears in the PRO group. The functional demand for rapid and accurate dorsiflexion likely places increased emphasis on the neuromuscular control of the TA, particularly during complex rhythm sequences like Zap-3. In contrast, amateur dancers may face greater challenges in maintaining ankle stability and foot precision during high-speed transitions, which could influence the effectiveness and could be associated with increased mechanical demands or injury risk during movement execution.

Although muscle activation and co-contraction differ between the dominant and non-dominant legs, bilateral symmetry remains crucial for overall coordination and stability during movements. Optimal bilateral symmetry provides stable support during complex dance movements, allowing the two legs to complement and coordinate with each other effectively [22,36]. The symmetry between the dominant and non-dominant legs is essential for achieving precision and stability in performance.

To our knowledge, only one study has used electromyography to record lower limb activity in professional dancers during the execution of flamenco footwork [11]. The previous study recorded the sEMG of an experienced professional dancer and described the patterns of changes in amplitude associated with the basic footwork of flamenco dance within a time domain [11]. This preliminary study provided the foundation for our research, which further explores muscle activation, muscle co-contraction and bilateral asymmetry. In our study, we involved a larger number of participants and considered the differences in proficiency levels among dancers. Additionally, the footwork test in our study was not executed in isolation; rather, it was performed within the context of Zap-3, which closely simulates a real choreography scenario.

This study has several limitations. Although the sample size of 12 dancers is larger than that in previous studies, it remains relatively small. Additionally, the use of mean/peak sEMG normalization limits the ability to compare activation amplitudes across groups or muscles. Future studies could address these issues by employing larger sample sizes, using maximum voluntary contraction (MVC) for normalization and incorporating ground reaction force and kinematic data to enhance understanding of external loading and joint mechanics.

## 5. Conclusions

The PRO dancers may employ task-specific neuromuscular strategies to optimize performance at maximum speed. While the present results do not establish a causal relationship between asymmetry and injury risk, understanding these asymmetries may help to inform individualized training strategies aimed at optimizing performance and reducing potential risks. These findings may also provide a basis for future studies investigating biomechanical mechanisms and preventive approaches in flamenco dance.

## Figures and Tables

**Table 1:** Descriptive characteristics of participants (N = 12).

Characteristics	Group PRO N = 6	Group AMT N = 6	*p* Value
Age (years)	38.83 ± 7.96	34.50 ± 10.67	0.148
Height (m)	1.67 ± 0.10	1.62 ±0.03	0.681
Mass (kg)	63.33 ± 6.38	56.17 ± 15.99	0.055
BMI (kg/m^2^)	22.79 ± 1.95	21.36 ± 6.00	0.078
Flamenco dance experience (years)	7.67 ± 4.89	1.83 ± 1.17	0.009 *

The professional group (PRO) and the amateur group (AMT); Body Mass Index (BMI); * denotes a significant difference between groups, *p* < 0.05.

**Table 2:** Foot strike frequency at different speed levels in professional (N = 6) and amateur dancers (N = 6).

Speed	160 BPM		180 BPM		F Speed	
Group	PRO	AMT	PRO	AMT	PRO	AMT
Frequency (Hz)	5.33	5.33	6.00	6.00	8.99 ± 0.78 *	7.08 ± 0.50

The professional group (PRO) and the amateur group (AMT); 160 bpm (160), 180 bpm (180), the fastest speed level (F); Hz: hertz; BPM: beats per minute; * denotes a significant difference between groups at *p* < 0.05.

**Table 3:** The sEMG normalized values of both the dominant and non-dominant leg in professional (N = 6) and amateur dancers (N = 6).

Muscle	Speed	sEMG Dominant Leg		sEMG Non-Dominant Leg	
		Group PRO	Group AMT	Group PRO	Group AMT
GM	160	3.93 ± 0.64	4.02 ± 2.37	6.33 ± 2.87	6.10 ± 1.16
	180	3.78 ± 0.59	3.61 ± 1.11	5.55 ± 1.71	5.39 ± 1.75
	F	4.27 ± 0.56 *	4.00 ± 1.62	6.95 ± 1.84	6.15 ± 2.52
BF	160	5.68 ± 2.28	6.60 ± 1.58	4.07 ± 0.84	5.91 ± 3.25
	180	7.26 ± 3.30	5.49 ± 2.56	4.54 ± 0.67	4.77 ± 2.64
	F	7.75 ± 2.47 *	5.36 ± 2.64	4.48 ± 0.32	5.38 ± 1.93
TA	160	7.35 ± 1.76	5.74 ± 3.10	6.67 ± 1.63	7.48 ± 1.99
	180	7.06 ± 1.82	5.31 ± 2.36	6.43 ± 1.60	5.31 ± 1.76
	F	7.58 ± 2.26	5.37 ± 1.81	8.69 ± 1.87	6.09 ± 2.64
RF	160	7.90 ± 1.33	9.09 ± 2.44	6.26 ± 2.82	6.76 ± 2.01
	180	7.40 ± 1.94	7.13 ± 1.99	6.44 ± 2.21	6.33 ± 2.79
	F	6.92 ± 1.48	6.60 ± 1.29	7.65 ± 2.03	6.93 ± 3.34

Gastrocnemius medialis (GM), biceps femoris in dominant leg (BF), tibialis anterior (TA), rectus femoris (RF), the professional group (PRO) and the amateur group (AMT), 160 bpm (160), 180 bpm (180), the fastest speed level (F). * Significant differences between dominant leg and non-dominant leg (*p* < 0.05, d > 1.67).

**Table 4:** Co-contraction index of dominant and non-dominant leg in professional (N = 6) and amateur dancers (N = 6).

Antagonist/Agonist	Speed	CCI Dominant Leg		CCI Non-Dominant Leg	
		Group PRO	Group AMT	Group PRO	Group AMT
GM/TA	160	0.58 ± 0.21	0.71 ± 0.23	0.98 ± 0.41	0.85 ± 0.24
	180	0.57 ± 0.19	0.77 ± 0.30	0.93 ± 0.44	0.87 ± 0.20
	F	0.63 ± 0.32	0.80 ± 0.35	0.82 ± 0.22	0.83 ± 0.19
BF/RF	160	0.72 ± 0.26	0.77 ± 0.24	0.76 ± 0.34	0.94 ± 0.53
	180	1.08 ± 0.66	0.78 ± 0.35	0.76 ± 0.21	0.67 ± 0.20
	F	1.14 ± 0.33 *	0.79 ± 0.30	0.62 ± 0.15	0.77 ± 0.27

Co-contraction index (CCI), Gastrocnemius medialis (GM), biceps femoris in dominant leg (BF), tibialis anterior (TA), rectus femoris (RF), the professional group (PRO) and the amateur group (AMT), 160 bpm (160), 180 bpm (180), the fastest speed level (F). * Significant differences between dominant leg and non-dominant leg (*p* < 0.05, d > 1.67).

## Data Availability

The data presented in this study is available on request from the corresponding author.
